# The genetic impact of heat stress on the egg production of Thai native chickens (Pradu Hang dum)

**DOI:** 10.1371/journal.pone.0281328

**Published:** 2023-02-03

**Authors:** Wipas Loengbudnark, Vibuntita Chankitisakul, Wuttigrai Boonkum

**Affiliations:** 1 Department of Animal Science, Faculty of Agriculture, Khon Kaen University, Khon Kaen, Thailand; 2 The Research and Development Network Center of Animal Breeding and Omics, Khon Kaen University, Khon Kaen, Thailand; University of Agriculture Faisalabad, PAKISTAN

## Abstract

Sustainable poultry production in adverse weather conditions is a widely debated issue, which has led to research into the development of breeds of poultry that are genetically resistant to heat. This study aimed to investigate the effects of heat stress on the genetics of monthly egg production and examine the threshold point of heat stress for preventing thermal stress and its effects on chicken productivity. The data of 5,965 monthly egg production records of 629 Thai native Pradu Hang dum chickens were used for analysis in combination with the temperature-humidity index (THI) calculated by meteorological data near the testing station. The average THI throughout the year was 76.6, and the highest was 82. The THI data were subsequently used to find the threshold point of heat stress. The THI equation used in this study was chosen by its highest correlation (-0.306) between THI values and monthly egg production. At a THI of 74, the lowest -2 logL was found and was considered the threshold point of heat stress. This means that monthly egg production would start decreasing when the THI was 74. Heritability was 0.15±0.03, and genetic and permanent environmental correlations were -0.29 and -0.48, respectively. The threshold point was used to estimate the estimated breeding values (EBVs) of the monthly egg production and heat stress individually, and EBVs were calculated into the selection index. The selection index values when the animal was selected for the replacement herd for all chickens (top 50%, 30%, 20%, and 10%) were 0.14, 0.90, 1.27, 1.53, and 1.91, respectively, and the genetic progress was 0.55, 0.60, 0.68, 0.75, and 0.77, respectively. This shows that the selection index values are lower if there are many selected animals. The recommendation for animal genetic selection is that the top 10% is appropriately because it seems to be most preferred. Therefore, using a selection index for high egg production and heat tolerance in Thai native chickens is possible to achieve genetic assessment in a large population.

## Introduction

The egg industry is one of the most crucial poultry industries that confront several challenges and obstacles worldwide. Therefore, there is an urgent need to keep improving hen’s genetic resistance and productive performance [1]. Heat stress effects directly impact the reduction of egg production, egg quality, chicken feed intake, and increased mortality in poultry [2–6]. Chickens raised in tropical areas are more likely to be affected by heat stress than those in temperate regions due to high temperatures and humidity throughout the year. Heat stress occurs when there is an imbalance between an animal’s body and ambient temperature, resulting in excessive heat load in the animal’s body until they cannot cool the heat out of their body [7–9]. Heat stress affects chickens in many ways; for example, behavioral and physiological changes lead to discomfort and can affect productivity [10–12]. When the core body temperature reaches a critical point (116.8°F or 47°C), which is called the lethal upper temperature, chickens can die from heat penetration [9]. Chicken farmers suffer economic losses [13–15], especially decreased egg production, which directly affects farmers’ incomes and is a major contributing factor to the global food shortage crisis [11, 16]. It was estimated by the world health organization (WHO) in 2021 [17] that as many as 8.9–10% of the world’s population were expected to suffer from hunger and malnutrition. Meanwhile, in 2022, the report of Global Report on Food Crisis 2022 (GRFC 2022), using the Global Hunger Index (GHI) to comprehensively measure and track hunger globally and by region and country, reported that around 193 million people in 53 countries/territories were suffering from severe food shortages and malnutrition [16]. Moreover, low egg production directly impacts the number of day-old chicks and increases production costs [8]. Therefore, increasing egg production during heat stress is a critical challenge in improving the productivity and profitability of chickens [18, 19].

Generally, the optimum temperature for performance is between 19–22°C for laying hens [20]. If the environment has a higher temperature and humidity, the chicken will also have a higher body temperature. Hence, they need to find a way to cool their body temperature. In nature, chickens use methods of ventilation such as panting [21, 22], increasing wing spreading [9], finding shelter from the heat [23], and drinking more water [24] to produce effects of evaporative cooling. At the same time, there are many approaches for the prevention of heat stress that can help chickens with heat dissipation, such as environmental and management adjustments (using the evaporative cooling system, mounting ventilation equipment, spraying water), lowering stocking density, better housing [11, 25, 26], feeding and nutrition management (diurnal feeding patterns, use of dietary supplements and minerals, and coarse particle and wet feeding) [4, 27–31]. In addition to those physiological responses, animal genetic selection methods (heat tolerance genes, QTL technique, and crossbreeding mating system between high-producing and native breeds) are alternative choices for sustainable selection [5, 19, 32–35].

The methods mentioned above are successful and give good results in commercial chickens. However, these methods are not long-term solutions, and they are exorbitantly expensive. One option that can provide lasting results is the genetic selection method of egg production under heat stress using the genetic model combined with the temperature-humidity index (THI). This genetic approach is widely used in dairy cattle, beef cattle, and pigs [36–40]. At the same time, the use of THI has been reported to study the relationship between heat stress effects and productivity characteristics in broilers and laying hens [41–43]; therefore, its application in native chickens in term of productivity and genetics is possible. THI is a value indicating the merged effects of air temperature and humidity correlated with the levels of heat stress, and it has been developed to monitor and reduce heat stress-related losses [44].

Native chicken breeds are significant for rural economies in several countries and are considered a genetic resource for developing high-yielding species [45, 46]. Native chickens are an inexpensive, high-quality protein source with good taste, good texture, and lower fat content, and are healthier than commercial broiler chickens [47, 48]. However, the research results on using quantitative genetics in the genetic selection of heat-tolerant native chickens are limited, and more information is needed.

Therefore, the objectives of this study were to investigate the effects of heat stress on phenotype and genetics for monthly egg production traits and to investigate the threshold point of heat stress in Thai native chickens.

## Materials and methods

### Data

The Institutional Animal Care approved all animals used in this study and the Use Committee of Khon Kaen University (No. IACUC-KKU-143/64). Data included 5,965 monthly egg production records of 629 Thai native chickens (Pradu Hang dum) obtained from the Network Center for Animal Breeding and Omics Research (NCAB), Faculty of Agriculture, Khon Kaen University. The experiment was conducted from March 2020 to February 2022. Pradu Hang dum is a Thai native chicken with black feathers, a black beak, and black shanks [49]. It has also been raised in all regions of Thailand for a long time.

The data were checked for normality by the Shapiro–Wilk test, for homogeneity of variance by the Levene’s test, and outlier data were eliminated before analysis. At birth, all chicks were leg tagged to assign their ID numbers and brooded for 4 weeks, then leg tags were removed, and wing tagging was used instead. They were vaccinated for infectious bronchitis, Newcastle disease, Fowlpox, and fowl cholera following the Network Center for Animal Breeding and Omics Research (NCAB) vaccination program. All chickens were reared in an open-housing system with an average of 12 h of natural light daily. They were fed with chicken commercial starter diet (19% crude protein, 2900 kcal ME/kg (ME = metabolizable energy)) for the brooding stage (first four weeks) and then changed to grower diet (15% crude protein, 2900 kcal ME/kg) from 4 weeks old to 20 weeks old with free access to fresh water. The chickens were morphologically considered (black feathers, a black beak, and black shanks) and selected at 12 weeks old of age, and chickens with disability were culled. At 20 weeks of age, the hens were moved to the battery cage (20 × 45 × 40 cm), and egg production was recorded starting when the chicken hen started laying the first egg (approximately 190 days of age) until 365 days of laying. During their laying period, hens were fed 100 g/d (17% crude protein, 2750 kcal ME/kg) for all assessments to fresh water.

Meteorological data, including daily air temperature (*T*) and relative humidity (*RH*), were obtained from the Thai Meteorological Department and recorded every 3 h at the Khon Kaen meteorological station, which is near NCAB (3 km from NCAB). The weather data were linked with the monthly egg production data and calculated into the THI according to four THI equations. After combining monthly egg production data and THI values, the statistical values were analyzed by Pearson correlation (r) to consider the most appropriate THI equations for the dataset. The forms of the THI equations are shown as follows.

Eq (1) by the National Ocean and Atmospheric Administration [50] is as follows:

$$THI=(1.8\times T_{avg}+32)-(0.55-0.0055\times RH_{Avg})\times (1.8\times T_{avg}-26)$$ (1)

where *T*_*avg*_ is the average air temperature (°C) and *RH*_*Avg*_ is the average relative humidity (%).

Eq (2) by Tao and Xin [51] is as follows:

$$THI=0.85(T_{max})+0.15(T_{min})$$ (2)

Eq (3) by Zulovich and DeShazer [52] is as follows:

$$THI=0.60(T_{max})+0.40(T_{min})$$ (3)

where *T*_*max*_ is the maximum air temperature (°C) and *T*_*min*_ is the minimum air temperature (°C).

Eq (4) cited by Marai et al. [53] is as follows:

$$THI=db^{\circ }\text{C-}\left[\left(0.31-0.31RH)(db^{\circ }\text{C}-14.4\right)\right]$$ (4)

where *db*°C is the dry bulb temperature in degrees Celsius and *RH* is the relative humidity percentage.

### Climate conditions

**Fig 1:**
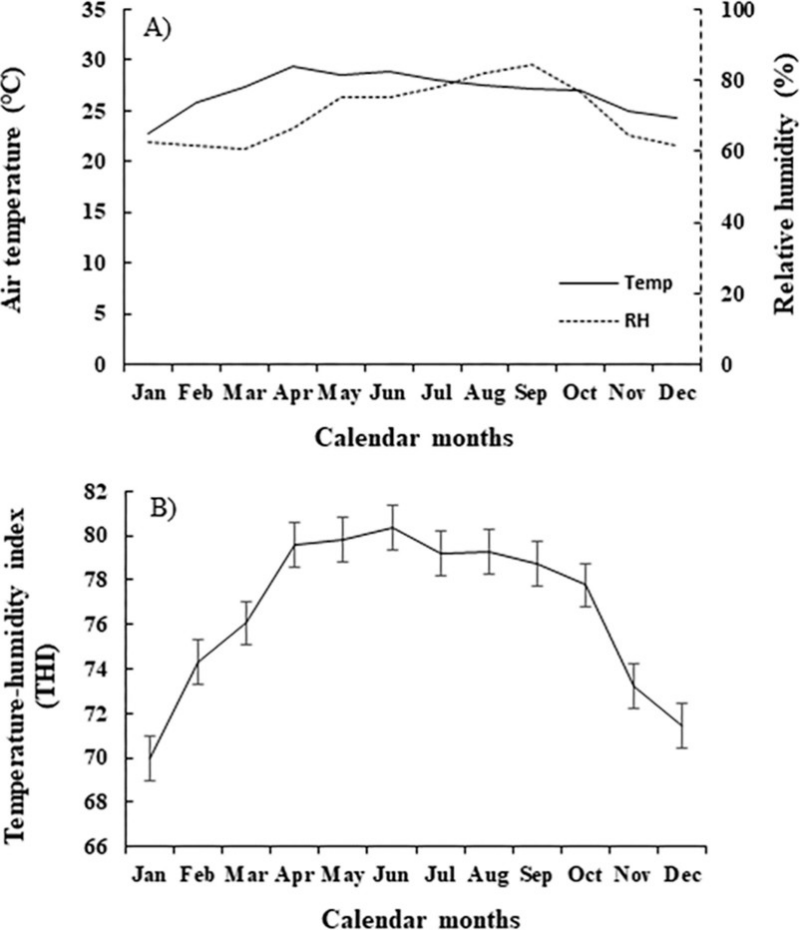
Average A) air temperature (—), relative humidity (----), and B) temperature-humidity index in Khon Kaen Province, Thailand, by calendar month during March 2020 to February 2022.

The climate condition of the research area (Khon Kaen Province; northeastern Thailand) during March 2020 to Feb 2022 could be characterized as hot and humid year-round. It was found that the average air temperature was 26.9°C. The highest temperature was in April (summer season), and the lowest temperature was in January (winter season). The relative humidity exceeded 67% for the entire year. The average THI was 76.6; the highest THI (82) was in April through July, and the lowest THI (68) was in January and December (Table 1 and Fig 1).

**Table 1:** Data structure for estimation of variance components for Thai native chickens.

Categories	N	Mean	SD	Max	Min
Animal with records (n)	629				
Animal with pedigrees (n)	866				
Number of records (n)	5,965				
Monthly egg production (eggs/month/bird)		12	7	27	1
Annual egg production (eggs/year/bird)		140	39	220	22
Age at first egg (days)		201	28	297	137
Air temperature (°C)		26.9	2.3	31.3	21.5
Relative humidity (%)		76.9	8.8	86.2	67.6
Temperature-humidity index, THI		76.6	3.8	82	68

### Genetic estimation

The repeatability test-day model was used to analyze the threshold point of heat stress, decrease in egg production according to THI level, estimated variance components, and genetic parameters (heritability, genetic correlation, and phenotypic correlation) using the Average Information Restricted Maximum Likelihood (AI-REML) algorithm [54]. The model is shown as follows:

$$y_{ijklm}=HG_{ij}+MIE_{k}AFE_{l}+\alpha (THI)+a_{0m}+a_{1m}[f(THI)]+p_{0m}+p_{1m}[f(THI)]+e_{ijklm}$$ (5)

where *y*_*ijklm*_ is the observation value of monthly egg production in hatch and generation (*HG*) class ij, months in egg (*MIE*) class k, age at first egg (*AFE*) covariate l, of animals m; *HG*_*ij*_ is the fixed effect of hatch and generation; *MIE*_*k*_ is the fixed effect of months in egg; *AFE*_*l*_ is the effect of age at first egg; *α*(*THI*) is the rate of decline of monthly egg production per a level of the temperature-humidity index (THI); *a*_0*m*_ is random additive genetic effects without consideration of heat stress of animal m; *p*_0*m*_ is random permanent environmental effects without consideration of heat stress of animal m; *a*_1*m*_[*f*(*THI*)] and *p*_1*m*_[*f*(*THI*)] are the random additive genetic and permanent environmental effects of animal m under heat stress conditions; and *e*_*ijklm*_ is the random residual effects.

The heat stress function is shown as follows [44]:

$$f(THI)=\left\{\begin{array}{l} 0\\ THI-THI_{threshold} \end{array}\begin{array}{l} ;THI\leq THI_{thrshold}(no\;heat\;stress)\\ ;THI>THI_{threshold}(heat\;stress) \end{array}\right.$$ (6)

The THI included in the repeatability test-day model was set at various critical values or threshold points. Different thresholds, at 70 (THI70), 72 (THI72), 74 (THI74), 76 (THI76), 78 (THI78), and 80 (THI80) of THI, were tested in the model. The best model was considered to come from the lowest minus twice the logarithm of the likelihood (−2logL) and the lowest Akaike information criterion (AIC).

Heritability (*h*^2^) was calculated as [44]:

$$h^{2}=\frac{\sigma_{a0}^{2}+\sigma_{a1}^{2}+2\sigma_{a01}}{\sigma_{a0}^{2}+\sigma_{a1}^{2}+2\sigma_{a01}+\sigma_{p0}^{2}+\sigma_{p1}^{2}+2\sigma_{p01}+\sigma_{e}^{2}}$$ (7)

Genetic correlations (*r*_*g*_) with and without consideration of heat stress effects of the additive genetic effects and correlations with and without consideration of heat stress effects of the permanent environmental correlations (*r*_*p*_) were calculated as follows [44]:

$$r_{g}=\frac{COV\sigma_{a0,a1}}{\sqrt{\sigma_{a0}^{2}*\sigma_{a1}^{2}}}$$ (8)

$$r_{p}=\frac{COV\sigma_{p0,p1}}{\sqrt{\sigma_{p0}^{2}*\sigma_{p1}^{2}}}$$ (9)

### Selection index

The selection index was calculated based on the estimated breeding values (*EBV*) for the traits, monthly egg production, and heat tolerance. The relative economic value (*ϑ* of each trait was a proportion of the standardized economic value to the total economic importance [55]. The trait of monthly egg production was determined to be more economically important than heat tolerance because egg production is directly related to production cost and benefit. Although heat tolerance is still essential, heat tolerance can also affect productivity due to physiological mechanisms. Therefore, the relative economic value of heat tolerance will be slightly lower than that of monthly egg production, at 0.70 and 0.30, respectively.

The selection index was calculated using the following formula [55]:

$$I=(\vartheta_{1}\times EBV_{1})-(\vartheta_{2}\times EBV_{2})$$ (10)

where *I* is the selection index value, *ϑ*_1_ = 0.70 and *ϑ*_2_ = 0.30 are relative economic values for monthly egg production and heat tolerance, respectively, and *EBV*_1_ and *EBV*_2_ are estimated breeding values for the traits.

Genetic progress using the formula is as follows [55]:

$$\Delta G=h^{2}\times SD$$ (11)

where Δ*G* is genetic progress, *h*^2^ is heritability, and *SD* is the selection differential between before and after selection.

## Results

### Determination of the appropriate THI equation and heat-stress threshold

The THI equations in this study were chosen based on the highest correlation value between THI values and monthly egg production, as shown in Table 2. The results showed that THI Eq 1 of the National Ocean and Atmospheric Administration (1976) had a significant and high negative correlation (-0.306) (p < 0.05) compared to the other THI equations; therefore, THI Eq 1 was used to estimate genetic parameters and to estimate breeding values for further selection index calculations. From Table 3, the lowest -2logL and AIC criteria were found at a THI of 74, which means that a THI of 74 was the threshold point of heat stress in this study and marks the beginning of a decline in egg production of Thai native chickens. To expand the THI of 74 in the form of air temperature and relative humidity, it was found that air temperatures in the range of 25.1 to 25.9°C and relative humidity in the range of 60.0 to 68.6% are considered the period when Thai native chickens begin to experience heat stress. At the same time, egg production began to decline with a drop of 0.32 eggs/THI of 74. Additionally, at a THI of 80, the drop in egg production was approximately 2 times higher than the THI of 74 (-0.67 egg/THI).

**Table 2:** Correlation and p value of four equations for calculating THI.

THI equations	Correlation (r)	p value
Equation1	-0.306	0.025
Equation1	-0.228	0.032
Equation1	-0.234	0.029
Equation1	-0.281	0.030

**Table 3:** Estimated variance components and genetic parameters for monthly egg production according to THI levels.

Parameters	THI70	THI72	THI74	THI76	THI78	THI80
$\sigma_{a0}^{2}$	5.87	5.85	**5.83**	5.80	5.80	5.80
$\sigma_{a1}^{2}$	0.02	0.02	**0.03**	0.10	0.23	0.41
*σ*_*a*0,*a*1_	-0.10	-0.10	**-0.12**	-0.23	-0.36	-0.49
$\sigma_{p0}^{2}$	7.95	7.93	**7.88**	7.87	7.87	7.87
$\sigma_{p1}^{2}$	0.28	0.32	**0.46**	1.84	4.14	6.36
*σ*_*p*0,*p*1_	-0.72	-0.77	**-0.92**	-1.95	-2.96	-4.12
$\sigma_{e}^{2}$	25.71	25.71	**25.75**	25.90	26.00	26.22
$\sigma_{total}^{2}$	38.19	38.09	**37.87**	37.15	37.40	38.44
*h*^2^(±*SE*)	0.15±0.02	0.15±0.02	**0.15±0.03**	0.15±0.03	0.14±0.04	0.14±0.04
*r*_*g*_	-0.29	-0.29	**-0.29**	-0.30	-0.31	-0.32
*r*_*p*_	-0.48	-0.48	**-0.48**	-0.51	-0.52	-0.58
**Model statistic criterion**						
-2logL	37414.42	37409.731	**37404.949**	37421.060	37421.060	37421.060
AIC	37428.42	37423.731	**37418.949**	37435.056	37435.056	37435.056
**Rate of decline of egg production (egg/bird/THI level)**						
*α*(*THI*)	0	0	**-0.32**	-0.46	-0.62	-1.22

$\sigma_{a0}^{2}$ = additive genetic variance without consideration of heat stress; $\sigma_{a1}^{2}$ = additive variance under heat stress conditions; *σ*_*a*0,*a*1_ = additive genetic covariance between effects with and without heat stress considered; *$\sigma_{p0}^{2}$* = permanent environmental variance without consideration of heat stress; $\sigma_{p1}^{2}$ = permanent environmental variance under heat stress conditions; *σ*_*p*0,*p*1_ = permanent environmental covariance between effects with and without heat stress considered; $\sigma_{e}^{2}$ = residual variance; $\sigma_{total}^{2}$ = total variance; *h*^2^(±*SE*) = heritability; *r*_*g*_ = genetic correlation; *r*_*p*_ = permanent environmental correlation; -2logL = minus twice the logarithm of the likelihood; AIC = Akaike information criterion; *α*(*THI*) is the rate of decline of monthly egg production per a level of temperature-humidity index (THI).

### Heat stress on variance components, heritability, and permanent environmental effects

The variance components and genetic parameters of the monthly egg production in Thai native chickens at various THIs are shown in Table 3. The additive genetic (5.80 to 5.87) and permanent environmental (7.87 to 7.95) variances without consideration of heat stress were not different in each THI value. In contrast, the additive genetic (0.02 to 0.41) and permanent environmental (0.28 to 6.36) variances under heat stress conditions were constant before the THI threshold, then increased after the THI threshold (THI of 74) and were many times greater when the THI was 80 (0.41 for additive genetic variance and 6.36 for permanent environmental variance). The estimates of heritability for monthly egg production ranged from 0.14 to 0.15. Moreover, the heritability values decreased with increasing THI.

### Heat stress on genetic and permanent environmental correlations

The genetic correlations with and without considering the heat stress effect (r_g_) at a THI of 74 showed a moderate negative correlation (−0.29) and were similar to other THI values and ranged from −0.29 to −0.32 (Table 3). These results can be described as high egg production having low heat tolerance. At the same time, the permanent environmental correlations with and without considering the heat stress effect (r_p_) at a THI of 74 were also negative (−0.48) and were similar to other THI values and ranged from −0.48 to −0.58.

### Selection index

**Fig 2:**
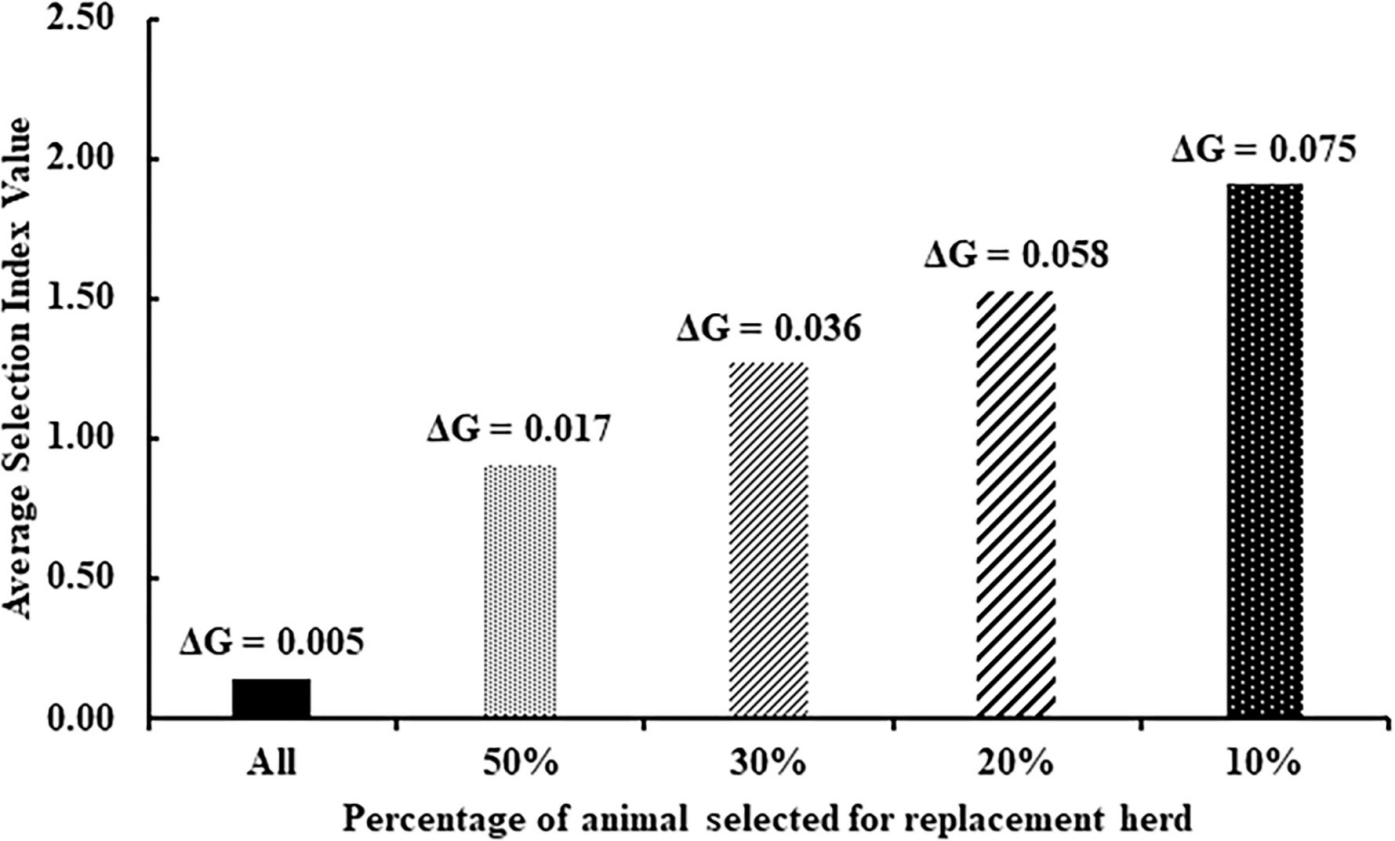
The selection index value (monthly egg number and heat tolerance) and genetic progress from all datasets and the top 50%, 30%, 20%, and 10% of the dataset in Pradu Hang dum chickens.

The selection index (monthly egg number and heat tolerance) was calculated, and the average selection index values for all chickens and the top 50%, 30%, 20%, and 10% are presented in Fig 2. The results indicated that the selection of native chickens that have good genetics of both high egg production and heat tolerance at the same time. The results also revealed that the higher the selection intensity, the higher the selection index, corresponding to genetic progress. The selection index values when the animal was selected for the replacement herd for all chickens (top 50%, 30%, 20%, and 10%) were 0.14, 0.90, 1.27, 1.53, and 1.91, respectively, and the genetic progress was 0.55, 0.60, 0.68, 0.75, and 0.77, respectively.

## Discussion

Heat stress is one of many factors affecting chicken productivity since it is induced by a negative balance between animal and environmental temperature, leading to unbalanced thermoregulation, their body’s cooling mechanisms becoming overwhelmed, and they might experience heat stress. Exposure to high ambient temperatures can decrease feed intake, which is the starting point of heat stress’s most detrimental effects on production and, consequently, the limitation of egg production due to insufficient nutrient supply [56]. At the same time, genetic improvement for high egg production can also lead to a genetic decline in animal thermotolerance due to the lack of consideration of the genetic improvement of both traits at the same time before.

### Threshold and egg production decline

This study showed that the threshold point of heat stress for monthly egg production in Thai native chickens (Pradu Hang dum) was at a THI of 74. In other words, egg production starts to decline if the air temperature was >25.9 degrees Celsius and the relative humidity was >68.6 percent. Kilic and Simsek [41], who used the THI equation by Zulovich and Deshazer [52], reported that when THI values increased from 25 to 29, egg production decreased by 25% in laying hens. In addition, Celik et al. [57] recommended that chickens avoid temperatures above 24°C, as this is the starting point of heat stress. Some reports have shown a detrimental effect on poultry productivity when temperatures exceed 30°C [58, 59]. The reduction in productivity results from behavioral and physiological changes in pursuit of balance thermoregulation, such as decreased feed intake, reduced uptake of available nutrients, and decreased digestibility of different components of the diet [2, 3]. This result is consistent with the report of Kim et al. [60], who studied the impacts of relative humidity on laying performance using three separate groups of laying hens. Each group was exposed to different relative humidity levels, low (25%), medium (50%), and high (75%), with high ambient temperature (30°C). The results showed that although relative humidity does not directly affect egg production, it affects feed intake, which is directly related to nutrient requirements and consequently affects egg production. Mignon-Grasteau et al. [61] performed their meta-analysis study regarding robustness to chronic heat stress in laying hens. Sensitivity to heat stress of shell strength, daily feed intake, egg mass, and hen-day egg production seemed more sensitive than the other traits, as it dropped approximately 13.6% between thermoneutral (15 to 20°C) and heat stress (30 to 35°C). Moreover, Deng et al. [62] studied a 12-day heat stress period that caused a 28.8% decrease in egg production. Moreover, acute heat stress (exposure to heat stress (35 ± 1°C) with relative humidity (55 ± 5%) for six hours) had a negative impact on egg production and quality in four genetically diverse strains of Egypt. Egg production dropped 17.65% in Lohman Brown, 23.21% in Golden Sabathia, 15.15% in Fayoumi, and 28.57% in White Leghorn [63]. Star et al. [64] reported a reduction of 36.4% in egg production and 3.41% in egg weight in laying hens subjected to heat stress. Additionally, heat stress has been shown to cause a significant reduction in egg weight (3.24%), eggshell thickness (1.2%), eggshell weight (9.93%), and eggshell percent (0.66%) [65]. Mashaly et al. [2] reported a marked decrease in egg production (28.8%), feed intake (34.7%), and body weight (19.3%) in laying hens subjected to chronic heat stress for 5 weeks. The observed reduction in feed intake may be caused by the hens’ attempt to maintain homeostasis by reducing their heat production [66]. Barrett et al. [5] found that heat stress lowered feed intake. Differences in animal breeds or genetic backgrounds and the quantity, severity, and duration of exposure to different heat stresses can explain the reported variation in the effects of heat stress. This could lead to the development of heat-tolerant poultry breeds specific to the area. In this study, the decline in egg production of Thai native chickens was 4.86% at a THI of 74 and 13.77% at a THI of 80, which was lower than previously mentioned. Important information for future genetic development is that chickens originated and were raised in tropical areas for a long time, like native chickens. Therefore, they are likely to better adapt to the environment than raise chickens from other sources in tropical regions [67, 68].

### Heritability

The heritability estimates of monthly egg production in this study ranged from 0.14 to 0.15. These estimates were similar to previous estimates reported by Tongsiri et al. [69] in Thai native chickens (Lueng Hang Kao Kabinburi) (0.15), Esfahan native chickens (0.16) [70], and Mazandaran native chickens (0.17) [71]. At the same time, the value from this study was different from many reported values, including in Horro chickens of Ethiopia (0.20 to 0.56) [72], in Korean Native Chickens (0.24 to 0.37) [73], and in Fayoumi chickens under conditions of heat stress (0.27 to 0.45) [19]. Yakubu et al. [74] also estimated a moderate h^2^ for Sasso egg production traits (h^2^ = 0.33) under tropical conditions.

One thing observed in this study is that heritability estimates tend to decrease with increasing THI values. The increase in THI values corresponds to an increase in additive genetic variance and permanent environmental variance under heat stress conditions, especially the permanent environmental variance, which will increase many times as the THI value increases, resulting in a decrease in heritability estimates. Additive gene effects for heat stress are comparable to heat shock protein (HSP) genes, which encode proteins involved in the cellular stress response. These proteins are called heat shock proteins because they are induced by exposure to elevated temperatures. They also play a role in the immune system, helping to protect cells from stress and damage, and are essential for maintaining cellular homeostasis and protecting against stress-induced damage [75]. These reasons explain why the additive genes are higher during heat stress in animals. Meanwhile, the permanent environmental variance would refer to the variations in the effects of heat stress on a population caused by differences in the environmental conditions that the individuals in the population experience rather than genetic differences. Factors contributing to the permanent environmental variance of heat stress effects include differences in the ambient temperature, humidity, and other climatic conditions that individuals are exposed. Besides, the differences in the type and intensity of physical and behavioral activity might affect their ability to cope with heat stress [35, 38, 76]. These results are in accordance with Boonkum et al. [46], who reported changes in variances and heritability of growth after the THI threshold point, which studied Thai slow-growing chickens (Kai Shee and Kaimook e-san1). It also showed a rise in variance changes and a decline in heritability.

However, the estimated heritability in this study was low (0.14 to 0.15), suggesting that genetic selection of heat tolerance and high egg production in chickens using the traditional method can result in low selection accuracy and slow selection progress [77]. For this reason, a combination of methods should be used to increase the accuracy of selection. For example, marker-assisted selection (MAS) is used in terms of preselection, and then the selection index is used for genetic selection in the final steps.

### Genetic and permanent environmental correlations

The genetic correlations with and without considering the heat stress effect were moderate negative correlations (−0.29). This indicated that selecting animal genetics for high egg production would lead to animal genetics with greater susceptibility to heat stress. Although the genetic correlation is negative, a low negative value may reveal the possibility of genetic improvement to enable chickens to have high egg production and heat tolerance using the selection index [44]. Another reason is that the genetic background of indigenous chickens is relatively heat-tolerant, resulting in less negative genetic correlation. Poultry has evolved to develop specific phenotypes that are useful for adapting to harsh environments wherever they live. Several phenotypes have been developed to mitigate heat stress effects, such as feather types, including naked neck, frizzle [78, 79], and plumage color [80, 81].

In addition, the permanent environmental correlations with and without considering the heat stress effect (r_p_) were also negative (−0.48). These values indicated that managing the environment for high egg production in Thai native hens would increase heat stress in chicken hens. In addition, the permanent environmental correlations were approximately two times higher than the genetic ones, meaning that chicken hens that respond to heat stress were expressed mainly by the environment rather than genetics. For this reason, to have higher egg productivity in harsh climates, optimum environmental improvements coupled with genetic improvements are the approaches to achieve better results, for example, an adjusted environment in terms of animal house pattern, ventilation system, natural or artificial shading, feed and feeding, water consumption [6, 11, 26].

### Selection index

The selection index was calculated using EBVs of the traits (monthly egg production and heat tolerance) and economic values. Values from the selection index also allow for balanced animal genetic selection decisions. It ranks the animals according to the overall genetic value of the traits to be genetically improved [82]. Genetic improvement occurs when genetic merit is improved through selection. The improvement in genetic merit refers to the overall improvement in a flock brought about by selection for several traits that contribute to the flock’s breeding objective. The present study presented the percentage of selection of animals for future replacement herds, which revealed that if many animals are selected, the values of the selection index will be lower as well. It is directly related to the increase in genetic progress [83]. On the other hand, selecting the animals too intensely (a small number of animals are selected) can result in higher inbreeding and decreased genetic variation [84]. Therefore, appropriate selection intensity or proportion matters to balance genetic improvement and inbreeding. The selection of the top 10% from the flock is preferable to other proportions since it is not too intense. As mentioned above, the genetic development of indigenous chickens to have great potential in heat tolerance and egg production can be achieved.

In conclusion, heat stress had a detrimental effect on both the reduction of egg production and the genetic decline of Thai native chickens. The impact of heat stress was evident at a THI of 74. In addition, selection for high egg production in native chickens would decrease heat tolerance in Thailand’s hot and humid climatic conditions. Therefore, the use of selection indices by the top 10% of selected birds showed that the combined genetic selection for high egg production and heat tolerance is possible and easy to implement in the bird’s genetic assessment of large populations.

## Supporting information

S1 File The egg production database is available doi: 10.17026/dans-x93-36wy. (XLSX)
